# Surgical Management of a Spinal Dural Arteriovenous Fistula

**DOI:** 10.7759/cureus.36533

**Published:** 2023-03-22

**Authors:** Joseph Yunga Tigre, Meredith C Costello, Krisna Maddy, Emily L Errante, Connor C Berger, Stephen S Burks

**Affiliations:** 1 Department of Neurosurgery, University of Miami Miller School of Medicine, Miami, USA; 2 The Miami Project to Cure Paralysis, University of Miami Miller School of Medicine, Miami, USA

**Keywords:** spinal cord diseases, spinal vascular malformation, arteriovenous fistula, spinal arteriovenous fistula, spinal dural arteriovenous fistulas (sdavfs)

## Abstract

Spinal dural arteriovenous fistulas (SDAVFs) may have subtle clinical presentations and are often misdiagnosed. Clinical status gradually deteriorates following symptom onset making prompt identification and management essential. Here we present a case of a 67-year-old patient with rapidly progressing motor and sensory deficits to eventual right hemiplegia. Following imaging and surgical intervention, a thoracic SDAVF was identified and resected. This case report highlights a unique SDAVF with a stroke-like presentation. For patients with such presentation, without a clear source of intracranial pathology, spinal causes such as SDAVF could be considered.

## Introduction

Spinal dural arteriovenous fistulas (SDAVFs) are acquired lesions that are low-flow arteriovenous shunts between a dural artery and the (peri)medullary venous system leading to venous hypertension and congestion [1]. SDAVFs may have subtle clinical presentations and are often diagnosed as more common spinal pathologies, such as degenerative diseases, myelopathies, or multiple sclerosis [2]. Identification and management of SDAVFs are essential as clinical status gradually deteriorates following symptom onset [1]. Here we present a case of an SDAVF in a 67-year-old patient as acute bilateral lower extremity weakness and sensory loss that rapidly progressed to right hemiplegia.

## Case presentation

Initial presentation

A 67-year-old, right-handed male with a past medical history of hypertension and lumbar spinal stenosis initially presented to our regional emergency department (ED) complaining of acute bilateral lower extremity weakness and sensory loss. While in the ED, his motor deficits progressed to complete right hemiplegia. The patient was noted to be well the evening prior. Upon waking, the patient was found on the floor by his bed, unable to get up due to weakness and with no sensation from the waist down. Symptoms then progressed to involve his right upper extremity. On exam in the ED, he was found to have right facial droop, 0/5 strength in his right upper and lower extremities, near normal strength (4+5) in the left upper extremity, and complete loss of sensation to his right hemibody. Prior to this event, the patient reported no history of sensory loss, stroke, myocardial infarction, or use of blood thinners. However, he did have a history of epidural steroid injections for chronic back pain. Upon further questioning, the patient endorsed a three-week history of gradual lower extremity weakness, although not limiting his ambulation, following his last injection.

Diagnostics

On initial physical examination, the patient was bradycardic and hypertensive (193/94), however he was alert and oriented to person, place, time, and location, with an NIH stroke scale score of 15. A stroke alert was called, given the patient’s symptomatology, and an initial brain computed tomography (CT) showed significant hemorrhage in the fourth ventricle extending into the central canal of the upper cervical spine. An intraparenchymal hemorrhage in the brainstem in the posterior lateral location was also found. He was started on a cardene drip and transferred to the ICU. Brain magnetic resonance imaging (MRI) and magnetic resonance angiography (MRA) confirmed the CT findings. Additionally, a cervical spine MRI showed abnormal T2 signal, likely representing vasogenic edema and blood products, in the medulla with extension into the cervical cord. The patient was then emergently transferred to our tertiary care hospital for further evaluation.

Following transfer, the patient was stable with an NIH stroke scale score of 15 but remained hypertensive. Initial neurosurgery evaluation revealed no indication for ventriculostomy or decompressive surgery. Tight blood pressure control recommendations were made, and the patient was admitted to the neurosciences intensive care unit (Neuro-ICU) for close monitoring and further workup.

**Figure 1:**
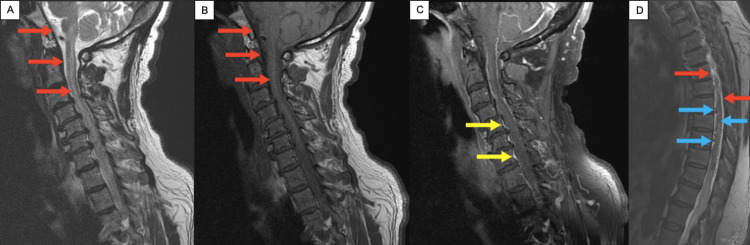
Progressive extension of intracranial hemorrhage into thoracic spine on MRIs The cervical (A, B, C) and thoracic spine (D) MRIs demonstrate the progressive extension of the hemorrhage down the spine. (A) is a sagittal T2-weighted image and (B) is a sagittal T1-weighted image of the cervical spine, with the red arrows demonstrating hypertense signal enhancements along the dural surfaces and intramedullary, thought to correspond to areas of blood. (C) is a sagittal T1-weighted image with gadolinium of the cervical spine, with the yellow arrows demonstrating contrast leaking into the spine. (D) is a sagittal T2-weighted image of the thoracic spine, with the red arrows further demonstrating hypertense signal enhancements along the dural surfaces and intramedullary. Here, the blue arrows also demonstrate serpiginous flow voids intradullary, most prominent seen from T5-10.

**Figure 2:**
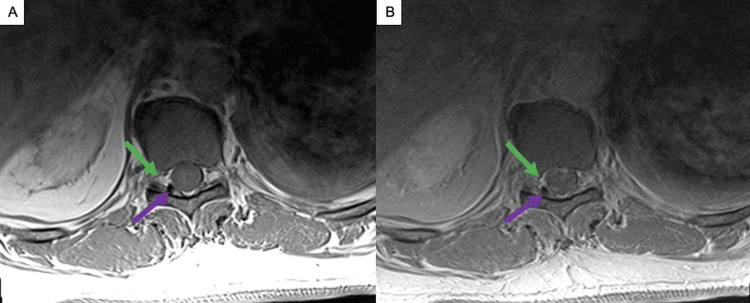
Arterial vascular fistula on thoracic spine MRIs The thoracic spine MRIs shown demonstrate the suggested arterial vascular fistula. (A) is an axial T1-weighted image without contrast and (B) is an axial T1-weighted image with contrast at the T10-11 level, with the green arrows demonstrating prominent areas of enhancement extending into the right T10-T11 foramen suggesting a dural AV fistula. On these axial views, the purple arrows also demonstrate the large, right-sided dorsal flow void seen.

Cerebral angiography was done and showed no evidence of an aneurysm, dissection, vascular malformation, or fistula. However, his cervical spine MRI showed his hemorrhage extending into the thoracic cord (Figure 1). Thoracic spine MRI suggested a spinal cord vascular malformation/arterial vascular fistula (AVF) (Figure 2). Specifically, prominent areas of dorsal enhancement at the T9 and T10 levels were seen, likely representing enlarged vessels. A spinal angiogram confirmed a dural AVF from the right T10 radicular artery with early venous drainage into the dilated epidural veins.

Intervention

**Figure 3:**
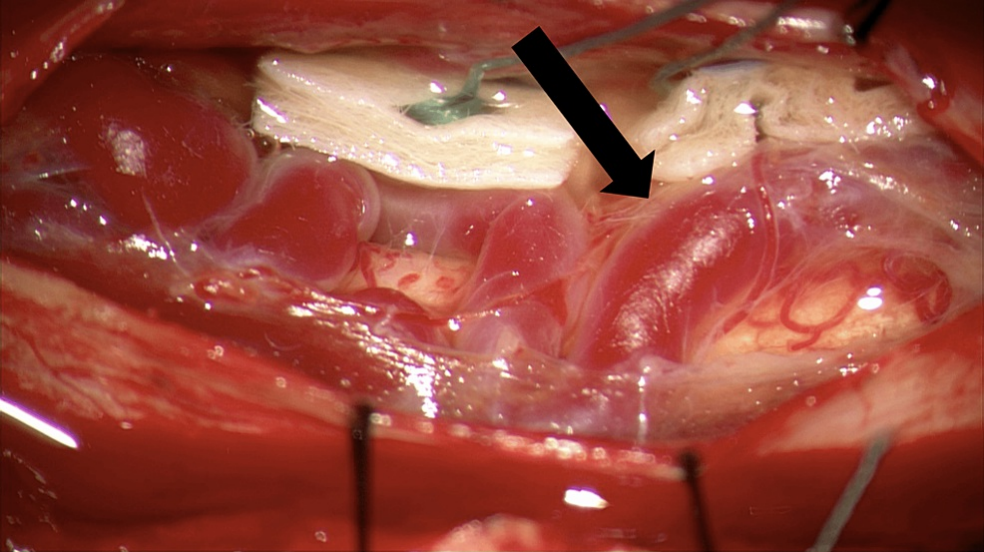
Initial exposure with severe venous dilation noted upon dural opening The intraoperative image shows the initial exposure of the dural. The black arrow demonstrates the severe venous dilation that obstructed the view of the spinal cord.

**Figure 4:**
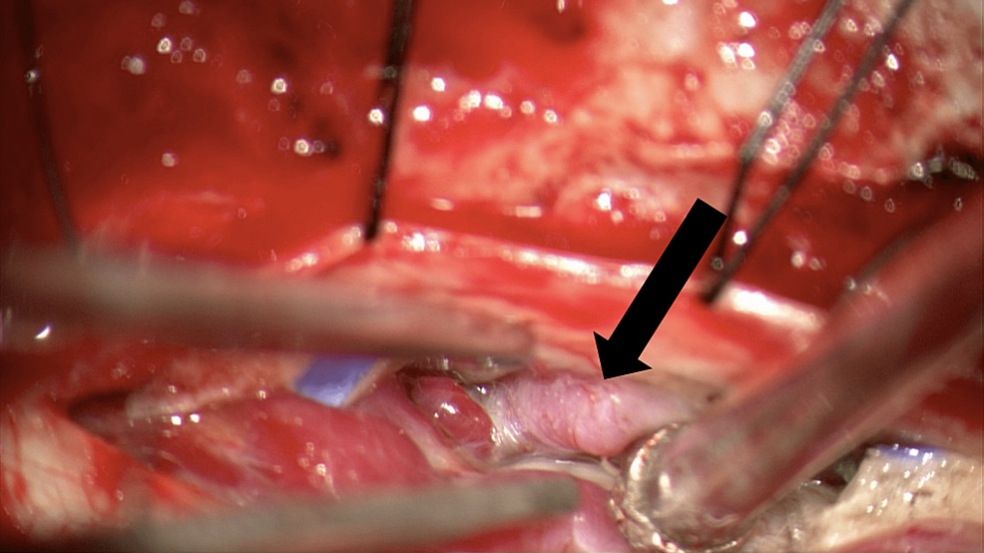
Arterialized vein fed from the dura superficial to the nerve root The intraoperative image shows dural reflection that allowed visualization of the arterialized vein. The black arrow demonstrates the arterialized vein fed from the dura superficial to the nerve root.

**Figure 5:**
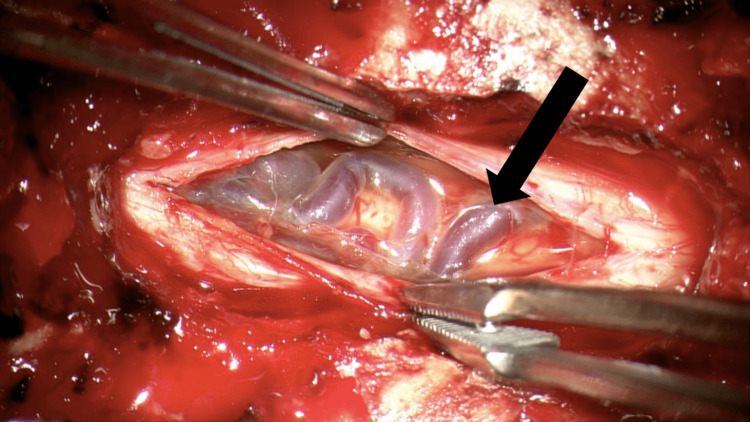
Dorsal veins changed in color and caliber after obliteration of the arterial vascular fistula (AVF) The intraoperative image shows the dorsal veins after obliteration of the AVF. The black arrow demonstrates that dorsal veins that are now changed in color (purple) and caliber (smaller).

After discussion with the patient and his family, a mutual decision was made to proceed with T9-T11 laminectomies and clipping of the dural AVF. The following day, the patient was brought into the operating room. After intubation and correct positioning, the T10 level was localized, and an incision was made centered over the pedicle. After T9-11 laminectomy, the dura was opened using the operative microscope via a paramedian incision. Intradurally, there was significant venous dilation, obstructing the view of the spinal cord (Figure 3). By reflecting the epidural veins and visualizing the exiting T10 nerve root, an arterialized vein fed from the dura superficial to the nerve root was identified (Figure 4). Further inspection confirmed this as the fistulous point. Upon placement of a temporary vascular clip, we noted a slight change in appearance to the dorsal veins supporting the diagnosis. Unfortunately, neuro-monitoring signals were not reliable, given the severity of his deficits. After several minutes the temporary clip was removed, and the vein was then coagulated and sectioned. With the arteriovenous (AV) connection obliterated, the dorsal veins changed in character and size, becoming purple and much smaller (Figure 5). The dura and wound were closed, and the patient was transferred back to the Neuro-ICU. Postoperatively, the patient’s exam did not change. He had severe bilateral lower and right upper extremity weakness, good strength in the left upper extremity, right hemi-body numbness, and normal mentation (GCS 15). After several weeks in the Neuro-ICU, due to difficulty weaning the ventilator, the patient was transferred to a long-term acute care facility with minimal neurologic improvement.

## Discussion

SDAVFs are rare, having an annual incidence of about 5-10 cases/1 million persons [2]. SDAVFs often have a nonspecific clinical presentation, presenting with low back pain, radiculopathy, lower extremity weakness, gait disturbances, paresthesias, and bowel/bladder disturbances. These symptoms are more frequently associated with more common spinal pathologies like spinal stenosis, degenerative disc disease, and peripheral neuropathy, leading to SDAVFs commonly being misdiagnosed [1]. In a retrospective study on avoiding the misdiagnosis of SDAVFs, Takai and Taniguchi found that 78% of their studied cohort (n=40) with SDAVFs were misdiagnosed, leading to additional disabilities and lack of significant improvement following correct treatment [3]. Previous studies have reported 12 to 44 months between symptom onset and diagnosis for SDAVFs; however, rapid deterioration has also been seen, most commonly due to hemorrhage [1]. Given the rarity of the rapidly deteriorating SDAVF presentation, it may not be initially considered highly on the differential.

In this case, given our patient’s deterioration to right hemiplegia, a stroke alert was called, and imaging revealed significant brain hemorrhage. Initially, it was assumed that the hemorrhage was isolated to the intracranial space. However, as a source could not be determined, further advanced imaging revealed progressive extension of the hemorrhage into the spine. Spinal angiogram ultimately confirmed the pathology of a dural AVF at T10. Although the correct diagnosis was ultimately made, additional time was required. As previously mentioned, the longer time needed to arrive at this accurate diagnosis may negatively impact patient outcomes [3]. 

Previous case reports and studies have highlighted rapidly deteriorating clinical SDAVFs presentations. However, in these reports, patients present with a loss of lower extremity motor function, sensory loss up to a specific dermatome, or gait disturbances, which help guide clinicians to a spinal etiology [4,5]. In our case, the patient presented with facial droop along with rapid onset of right hemiplegia leading to cranial etiology being initially suspected. SDAVFs with unique presentations represent a diagnostic challenge. Our case brings attention to this rare presentation and highlights the importance of comprehensive differentials in similar cases.

## Conclusions

SDAVFs are commonly misdiagnosed, and delayed time to correct diagnosis can negatively impact patient outcomes. Our case highlights a rare SDAVF presentation of rapid deterioration and hemiplegia. For similar patients with a stroke-like presentation without a clear source of intracranial hemorrhage, spinal causes such as SDAVF should be considered.
